# Does Statin Therapy Have a Beneficial Effect on Knee Osteoarthritis?

**DOI:** 10.7759/cureus.79420

**Published:** 2025-02-21

**Authors:** Abla El Hajjaji, Nessrine Akasbi, Imane El Mezouar, Taoufik Harzy

**Affiliations:** 1 Rheumatology, Hassan II University Hospital, Faculty of Medicine, Pharmacy and Dental Medicine, Sidi Mohamed Ben Abdellah University, Fez, MAR

**Keywords:** cardiovascular comorbidities, cardiovascular prevention, dyslipidemia, knee osteoarthritis (ko), microcirculation and inflammation, statins, visual analog scale (vas)

## Abstract

Introduction: Knee osteoarthritis (KO) is a major public health issue, that significantly affects patients' quality of life. Metabolic comorbidities, particularly dyslipidemia, have been shown to influence the incidence and progression of KO. Given the potential impact of hypercholesterolemia on disease progression, the hypothesis arises that dyslipidemia treatments, such as statins, may offer a beneficial effect in mitigating KO progression.

Objective: This study evaluates the effect of statin therapy on the clinical improvement of knee osteoarthritis (KO), particularly in terms of pain reduction, measured using the Visual Analog Scale (VAS).

Methods: A cross-sectional, retrospective, descriptive, and analytical study was conducted at the rheumatology department of the University Hospital Center Hassan II of Fez, between January 2018 and January 2024. Patients diagnosed with symptomatic primary knee osteoarthritis (KO) were recruited from the department's database of rheumatology consultations, and all were regularly followed up within the department. The patients were divided into two groups: Group 1, which received statin therapy, and Group 0, a control group that did not receive statins. The primary outcome was clinical improvement, which was assessed using the Visual Analog Scale (VAS) to measure pain intensity.

Results: A total of 465 patients were included, with 48 (10.3%) receiving statins. A significant clinical improvement in KO was observed in 34 (70.8%) patients in the statin group, compared to 196 (47.1%) patients in the control group (p=0.001). The analysis indicated that statin use was significantly associated with clinical improvement, independent of other demographic and comorbid factors.

Conclusions: Statin use may provide benefits in the management of KO, particularly in reducing pain, in addition to conventional symptomatic treatments. These results highlight the need for further prospective research to clarify the mechanisms of action of statins in this condition.

## Introduction

Knee osteoarthritis (KO), the most prevalent joint pathology, poses a significant public health burden due to its impact on patients' quality of life [1]. This condition arises from the interaction of local and systemic risk factors, with two primary pathophysiological mechanisms: the "mechanical" mechanism, associated with excessive joint load, and the "systemic" mechanism, involving inflammatory mediators such as adipokines and cytokines, primarily produced by adipose tissue [1].

Osteoarthritis associated with metabolic disorders exemplifies this interaction. Several studies have demonstrated the cumulative effect of metabolic comorbidities, including abdominal obesity, hypertension, diabetes, and dyslipidemia, on the incidence and progression of KO [2]. The relationship between metabolic syndrome and KO is well-established [3]. Dyslipidemia, particularly hypercholesterolemia, has been identified as a key risk factor in the progression of KO [4]. Research indicates that lipid imbalances correlate with radiological changes in KO, either early or late in disease progression [5].

In light of these findings, the hypothesis that dyslipidemia treatments, particularly statins, may influence the progression of KO has been proposed [6]. Statins, which inhibit HMG-CoA reductase, are widely used to manage hypercholesterolemia and reduce cardiovascular risk. However, the impact of statins on KO remains debated, with some studies suggesting potential therapeutic benefits and others pointing to their pleiotropic effects, which may be deleterious [7-9].

This study aims to evaluate the effect of statins on the clinical improvement of KO, as measured by the Visual Analog Scale (VAS) in patients with symptomatic primary KO.

## Materials and methods

Study design

This cross-sectional, retrospective, descriptive, and analytical study was conducted at the rheumatology department of the University Hospital Center Hassan II in Fez between January 2018 and January 2024. Patients with symptomatic primary knee osteoarthritis (KO) were recruited from the department’s database of rheumatology consultations, where they were regularly followed up.

Inclusion Criteria

The study included only patients with knee osteoarthritis at stage II (definite osteophytes with possible joint space narrowing) and stage III (moderate multiple osteophytes, definite joint space narrowing, some sclerosis, and possible bone end deformity) according to the Kellgren and Lawrence classification, as confirmed by standard weight-bearing radiographs.

Patients were divided into two groups: Study group (G1, patients receiving statin therapy); and Control group (G0, patients not treated with statins). The three types of statins prescribed in our cohort were simvastatin, atorvastatin, and rosuvastatin. However, the exact frequency of each statin type was not available in our database. Consequently, we did not perform a comparative analysis of the efficacy of different statins in knee osteoarthritis management. Statins were prescribed by endocrinologists and/or cardiologists for the management of dyslipidemia in the primary, secondary, or tertiary prevention of cardiovascular diseases. The choice of statin was based on the patient's cardiovascular risk profile and physician recommendations.

The primary inclusion criterion for G1 was regular statin use for at least one year for indications such as dyslipidemia, cardiovascular disease, or prevention of coronary artery disease recurrence. Both groups received symptomatic treatment, including analgesics (same dosage for both groups) and symptomatic slow-acting drugs for osteoarthritis (SySADOA). NSAIDs were used only for acute flare-ups, with a maximum duration of one week.

Exclusion Criteria

Patients were excluded if they had received hyaluronic acid or platelet-rich plasma injections, had undergone prosthetic surgery, had chronic inflammatory rheumatism, had secondary causes of KO, were non-compliant, or were lost to follow-up.

Outcome assessment and statistical analysis

Pain levels, as recorded in our institution’s patient registry, were retrospectively extracted. In routine clinical practice, pain was assessed using the Visual Analog Scale (VAS) at the first consultation and at three-month intervals during follow-up for a minimum duration of one year. Clinical improvement was the primary outcome measure. The study was conducted in three phases: descriptive phase in which collection of clinical, epidemiological, and sociodemographic characteristics was done; bivariate analysis in which assessment of associations between statin use and all study variables was carried out; multivariate analysis in which logistic regression analysis using SPSS software (IBM Corp 2017, IBM SPSS Statistics for Windows, Version 25.0.) to evaluate the influence of multiple factors on clinical outcomes was performed. Statistical analysis was performed using the student’s t-test for continuous variables and the chi-square test for categorical variables. Multivariate analysis was conducted using logistic regression, with statistical significance set at p<0.05.

## Results

A total of 465 patients were included in the study, with a sex ratio of 9.7 females to males and a mean age of 59.3 ± 10.8 years. The mean BMI for the total study population was 29.3 ± 4.5 kg/m². The average duration of knee osteoarthritis (KO) was 5.6 ± 3.7 years. The majority of patients (368, 79.1%) had a low-to-middle socioeconomic status. Cardiovascular risk factors included 6 (1.3%) smokers, 202 (43.4%) sedentary individuals, 109 (23.4%) diabetics, 119 (25.6%) hypertensives and 53 (11.4%) dyslipidemic patients. The most frequent form of KO was femorotibial medial (424, 91.2%), followed by femorotibial lateral (147, 31.7%) and femoropatellar involvement (118, 25.3%). Multicompartmental osteoarthritis was observed in 164 (35.2%) patients with co-occurring spinal and digital osteoarthritis in some cases.

The average Visual Analog Scale (VAS) score for KO pain was 5.4 ± 2.0. Of the patients, 95.4% (441 patients) were using as-needed analgesics, 92.1% (428 patients) were receiving slow-acting anti-arthritic drugs, and 48.8% (227 patients) had previously used short-term non-steroidal anti-inflammatory drugs (NSAIDs).

The study group (G1) comprised 48 patients (10.3% of the sample) on statins. Among these, 70.8% showed clinical improvement in KO. The control group (G0) included 417 patients (89.7%), of whom 47.1% showed clinical improvement. A statistically significant reduction in pain was observed in the statin group (G1) from six months onwards. The evolution of VAS scores over time for both groups is summarized in Table 1.

**Table 1:** Evolution of VAS Scores over follow-up.

Follow-up time	Statin group (G1) (N=48)	Control group (G0) (N=417)	p-value	t-value
Baseline	5.5 ± 2.1	5.3 ± 1.9	0.12	1.56
3 months	4.3 ± 1.8	4.9 ± 2.0	0.045	2.01
6 months	3.8 ± 1.7	4.5 ± 1.9	0.03	2.25
9 months	3.4 ± 1.6	4.2 ± 1.8	0.025	2.35
12 months	3.1 ± 1.5	4.0 ± 1.7	0.015	2.57

Bivariate analysis revealed that statin use was significantly associated with improvement in KO (p=0.001) (Table 2). Cardiovascular and metabolic comorbidities, including hypertension and diabetes, were prevalent in both groups, with no significant differences between the statin group (G1) and the control group (G0). This suggests that the observed improvement in the statin group is unlikely to be attributed to the presence of these comorbidities. The mean age, BMI, initial VAS score, disease duration, and treatments administered were similar between both groups, ensuring that the effects of statins could be assessed independently of these variables. In terms of clinical improvement, 70.8% of patients in the statin group (G1) showed significant improvement in knee osteoarthritis, compared to 47.1% in the control group (G0). This difference was statistically significant (p=0.001), supporting the hypothesis that statins may offer a beneficial effect in the management of KO.

**Table 2:** Characteristics of knee osteoarthritis patients treated or not with statins over the 6-year study period (N=465)

Variable	Study group (G1: statins) (N=48)	Control group (G0: No statins) (N=417)	p-value	Chi-square value
Sex (F/M) N (%)	9 (18.8%), 39 (81.2%)	58 (13.9%), 359 (86.1%)	0.32	0.98
Mean age (years)	59.5 ± 10.2	59.2 ± 10.6	0.74	N/A
BMI (Kg/m²)	29.7 ± 4.3	29.2 ± 4.6	0.58	N/A
Mean duration of KO (years)	5.3 ± 3.0	5.7 ± 3.8	0.48	N/A
Hypertension (%)	12 (25.0%)	114 (27.3%)	0.68	0.56
Diabetes (%)	6 (12.5%)	92 (22.1%)	0.1	2.7
Dyslipidemia (%)	8 (16.7%)	46 (11.0%)	0.3	1.89
Sedentary lifestyle (%)	20 (41.7%)	179 (42.9%)	0.91	0.01
Initial VAS (mean ± SD)	5.5 ± 2.1	5.3 ± 1.9	0.12	N/A
Clinical improvement of KO	34 (70.8%)	196 (47.0%)	0.001	10.25

Furthermore, multivariate logistic regression analysis confirmed that statin use was an independent predictor of clinical improvement (OR=2.12, 95% CI: 1.32-3.41, p=0.002), after adjusting for age, sex, BMI, and metabolic comorbidities. The detailed results of the logistic regression analysis are presented in Table 3.

**Table 3:** Logistic regression analysis for clinical improvement in KO. Statin use is an independent predictor of clinical improvement in KO (OR=2.12, 95% CI: 1.32-3.41, p=0.002), after adjusting for age, sex, BMI, and metabolic comorbidities.

Variable	Odds ratio (OR)	95% Confidence interval (CI)	p-value
Statin use (G1)	2.12	1.32-3.41	0.002
Age	1.05	0.98-1.12	0.15
Sex (female)	1.20	0.85-1.68	0.30
BMI	1.08	0.95-1.21	0.22
Hypertension	0.92	0.68-1.35	0.45
Diabetes	0.88	0.62-1.27	0.50
Dyslipidemia	1.15	0.77-1.64	0.35

## Discussion

Knee osteoarthritis (KO) is a degenerative joint disease characterized by cartilage degeneration, subchondral bone remodeling, and synovial membrane inflammation. It primarily affects older adults, leading to chronic pain, functional impairment, and a reduced quality of life. Despite the availability of symptomatic treatments, including analgesics, non-steroidal anti-inflammatory drugs (NSAIDs), intra-articular hyaluronic acid, and platelet-rich plasma (PRP) injections, there are currently no therapies that halt disease progression. As such, exploring novel therapeutic strategies is critical. The present study evaluates the potential beneficial effects of statins in the management of gonarthrosis, providing evidence that statins may improve clinical outcomes in KO patients.

Our findings suggest that statin use is associated with significant clinical improvement in knee osteoarthritis, particularly in pain relief, independent of other confounding factors such as the use of symptomatic slow-acting drugs for osteoarthritis (SySADOA), NSAIDs, hyaluronic acid, PRP injections, disease duration, and initial symptom severity. This improvement, measured by the Visual Analog Scale (VAS), reflects a meaningful enhancement in patient-reported outcomes, which are central to the management of KO. Although clinical symptoms and radiological findings in KO often dissociate, our results indicate that statin use is linked to a clear clinical benefit, supporting the hypothesis that statins may have a direct effect on the progression of the disease.

Experimental studies have suggested that statins, through inhibition of HMG-CoA reductase, may protect articular cartilage by modulating inflammatory responses and extracellular matrix components. Research in animal models, including murine and rabbit studies, has shown that statins administered both orally and intra-articularly can reduce cartilage degradation, likely through the downregulation of inflammatory factors [10-12]. Specifically, Baker et al. (2010) demonstrated that statins could modulate the expression of catabolic and inflammatory genes in human chondrocytes, indicating a direct anti-inflammatory effect on cartilage [7].

The anti-inflammatory properties of statins are particularly relevant in the context of KO. Several studies have shown that statins can decrease the expression of pro-inflammatory cytokines, such as TNF-α and IL-6, as well as prostaglandins in synovial fluid and joint tissues [13-18]. Saberianpour et al. (2022) highlighted that statins inhibit the production of inflammatory cytokines and reduce the activation of pro-inflammatory pathways in cartilage cells, potentially alleviating pain and improving joint function [8]. Clinical reports have also shown that patients taking statins for cardiovascular conditions often experience a reduction in musculoskeletal pain, suggesting that statins may benefit individuals with KO by mitigating inflammation and associated pain [19-22].

Our study assessed clinical improvement using the Visual Analog Scale (VAS), a widely used tool for measuring pain severity in knee osteoarthritis (KO). While effective in clinical practice, the VAS has limitations in sensitivity and specificity, particularly for long-term treatment outcomes. Additionally, patient-reported pain levels may not always correlate with radiological findings, as clinical symptoms and disease progression in KO are often dissociated [23]. We acknowledge that relying solely on the VAS has its limitations and that more comprehensive and efficient scoring systems would be ideal. However, due to the retrospective design of our study and the use of pre-existing registry data, the VAS was the most accessible measure, systematically recorded for all patients. Despite its limitations, it provides a reliable basis for our analysis and highlights the need for future prospective studies incorporating radiographic assessments and functional evaluations to complement patient-reported outcomes.

Similar studies have reported positive effects of statins in other forms of osteoarthritis. Zhang et al. (2022) found that statin treatment led to significant reductions in pain and improvements in joint function in patients with hip osteoarthritis. Additionally, they noted a reduction in inflammatory markers in synovial fluid, further supporting the anti-inflammatory role of statins in osteoarthritis management [24]. Sarmanova et al. (2020) also reported that statin use was associated with a reduced risk of joint replacement due to osteoarthritis and rheumatoid arthritis, as demonstrated in their propensity-score matched longitudinal cohort study [25].

However, our study has several limitations. The three types of statins prescribed in our cohort were simvastatin, atorvastatin, and rosuvastatin. However, the exact frequency of each statin type was not available in our database. Consequently, we did not perform a comparative analysis of the efficacy of different statins in knee osteoarthritis management. Pain assessment, while highly relevant, is subjective and may introduce bias. We also did not account for variables such as treatment adherence or changes in statin dosage, which could affect the observed outcomes. Moreover, the effects of statins on other critical aspects of KO, such as joint function or radiological progression, were not evaluated. These are areas for future research, where investigating the effects of statins in combination with other anti-inflammatory or cartilage-protective therapies, like SAARDs, would be valuable. Additionally, a more personalized approach potentially incorporating genetic profiling could identify patients most likely to benefit from statins based on individual variations in drug metabolism.

On the whole, our study provides evidence that statins may offer a beneficial clinical effect in the management of knee osteoarthritis, particularly in pain relief. These findings support further investigation into the role of statins as a potential adjunctive therapy for KO, particularly in combination with other disease-modifying treatments. Future research should address the long-term impact of statins on disease progression, joint function, and radiological outcomes.

## Conclusions

The results of our study suggest that statin use in the management of knee osteoarthritis may not only reduce cardiovascular risk but also improve clinical symptoms, particularly pain, associated with the disease. These findings warrant further prospective interventional studies to elucidate the mechanisms underlying statin effects and their potential role in the management of knee osteoarthritis. Additionally, future research should investigate whether the benefits observed vary depending on the specific statin molecule used, which could provide valuable insights for optimizing treatment strategies.
